# Declining Burden of *Plasmodium vivax* in a Population in Northwestern Thailand from 1995 to 2016 before Comprehensive Primaquine Prescription for Radical Cure

**DOI:** 10.4269/ajtmh.19-0496

**Published:** 2019-11-18

**Authors:** Cindy S. Chu, Verena I. Carrara, Daniel M. Parker, Stéphane Proux, Prakaykaew Charunwatthana, Rose McGready, François Nosten

**Affiliations:** 1 Shoklo Malaria Research Unit, Mahidol–Oxford Tropical Medicine Research Unit, Faculty of Tropical Medicine, Mahidol University, Mae Sot, Thailand;; 2 Centre for Tropical Medicine, Nuffield Department of Medicine, University of Oxford, Oxford, United Kingdom;; 3 Department of Medicine, Swiss Tropical and Public Health Institute, Basel, Switzerland;; 4 Department of Population Health and Disease Prevention, University of California, Irvine, California;; 5 Mahidol–Oxford Tropical Medicine Research Unit, Faculty of Tropical Medicine, Mahidol University, Mae Sot, Thailand;; 6 Department of Clinical Tropical Medicine, Faculty of Tropical Medicine, Mahidol University, Bangkok, Thailand

## Abstract

All *Plasmodium* cases have declined over the last decade in northwestern Thailand along the Myanmar border. During this time, *Plasmodium vivax* has replaced *Plasmodium falciparum* as the dominant species. The decline in *P. falciparum* has been shadowed by a coincidental but delayed decline in *P. vivax* cases. This may be due to early detection and artemisinin-based therapy, species-specific diagnostics, and bed net usage all of which reduce malaria transmission but not *P. vivax* relapse. In the absence of widespread primaquine use for radical cure against *P. vivax* hypnozoites, the decline in *P. vivax* may be explained by decreased hypnozoite activation of *P. vivax* relapses triggered by *P. falciparum*. The observed trends in this region suggest a beneficial effect of decreased *P. falciparum* transmission on *P. vivax* incidence, but elimination of *P. vivax* in a timely manner likely requires radical cure.

## INTRODUCTION

Malaria has decreased in the Greater Mekong Subregion (GMS) over the last decade. The WHO estimates that from 2012 to 2015, malaria cases declined by 54% and malaria-related mortality by 84% in this region. All GMS nations have drafted plans for malaria elimination by 2030.^1^ Because of the emergence of artemisinin-resistant parasites *Plasmodium falciparum* rather than *Plasmodium vivax* has been the focus of elimination. *Plasmodium vivax* remains a major contributor to morbidity largely because of relapses (as high as 80% in northwest Thailand).^2^ Treating relapses (radical cure) requires completing a prolonged primaquine course (7–14 days), leading to decreased adherence. Daily primaquine doses (0.25–0.5 mg/kg/day) needed for radical cure are contraindicated in persons with glucose-6-phosphate dehydrogenase (G6PD) deficiency because of hemolytic risk.^3^ When G6PD testing is not available, primaquine prescription is less likely. A weekly (for 8 weeks) regimen is recommended for G6PD-deficient individuals, which also limits adherence. Currently, primaquine is contraindicated during pregnancy and lactation.^3^ Consequently, the drug is often not prescribed, relapses are not prevented, and clinical infections recur after schizonticidal treatment.

*Plasmodium falciparum* malaria has decreased along the Thailand–Myanmar border over the last decade.^4^ Without widespread radical cure, a different pattern might be expected for *P. vivax*. Here, we report longitudinal trends in passively detected *P. vivax* infections in medical clinics along the Thailand–Myanmar border for a period spanning 22 years.

## METHODS AND RESULTS

Shoklo Malaria Research Unit (SMRU) operates clinics located in Thailand along the Moei River, which forms the border with Myanmar. The unit has field-based medical clinics, situated 30–120 km outside Mae Sot, Thailand, serving refugee and migrant (mostly unregistered) border populations. The refugee clinic operated from 1986 to 2016. The first migrant clinic was opened in 1998, second in 1999, and two additional clinics were opened in 2004–2005.

Malaria transmission in this area is low, unstable, and seasonal.^5^ The dominant species are *P. falciparum* and *P. vivax*, and *Plasmodium malariae* and *Plasmodium ovale* are uncommon. In patients with a fever history, a malaria smear was performed routinely in the refugee clinic, whereas in the migrant clinics malaria rapid diagnostic tests (RDTs) were also performed. Malaria smears in the migrant clinics were performed systematically in children aged < 5 years and during a one-week period each month (called “smear week”).^6^ Before March 2010, the RDTs that were used diagnosed only *P. falciparum* so *P. vivax* was a clinical diagnosis (i.e., fever and RDT negative) during the non “smear weeks”. When newer RDTs were used, all *P. vivax* diagnoses were confirmatory (RDTs or malaria smear). Malaria microscopy was performed by trained laboratory technicians. Standardized quality control for malaria smear and RDT reading was conducted routinely.

At SMRU, mefloquine–artesunate was the first-line treatment for *P. falciparum* and mixed *P. falciparum* infections from 1994 until June 2012 when it was changed to dihydroartemisinin–piperaquine plus gametocytocidal single-dose primaquine. Chloroquine has remained efficacious against *P. vivax*. After 2010, routine radical cure for *P. vivax* was implemented, but only under direct supervision, resulting in < 10% of patients with *P. vivax* treated with primaquine. Until 2017, there were no targeted radical curative interventions in the population. However, from 2010 to 2014, a series of *P. vivax* studies were performed and primaquine was prescribed to ∼1,200 participants. A *P. falciparum* elimination campaign in community-based malaria clinics using standard artemisinin-based therapy (ACTs) and single-dose primaquine began in 2014 in Kayin State, Myanmar, covering areas opposing Tak Province. In these community-based malaria clinics, some overlapping the SMRU catchment area, radical cure for *P. vivax* was not prescribed.^7,8^

For this analysis, aggregated anonymized data from monthly reports on nonpregnant patients with *P. falciparum* and *P. vivax* malaria diagnosed in the SMRU medical clinics from July 1995 to December 2016 in refugees and November 1998 to December 2016 in migrants (before universal primaquine administration) were included. Mixed infections were attributed to both *P. falciparum* and *P. vivax* cases. In years with clinical *P. vivax* diagnosis, the annual caseload was adjusted by the proportion of *P. vivax* cases detected during the “smear weeks” of the same year. Descriptive statistics were used for *P. vivax* and *P. falciparum* cases by year, age (grouped every 5 years until 39 years, then all ≥ 40 years), gender, and status (migrant or refugee). Poisson regressions were used to test for differences in trends in cases over time by age-group, gender, and status. Then the data were stratified by gender with an interaction term between year and age-group to detect changing patterns in the distribution of cases across the lifespan, over time.

**Figure 1.**
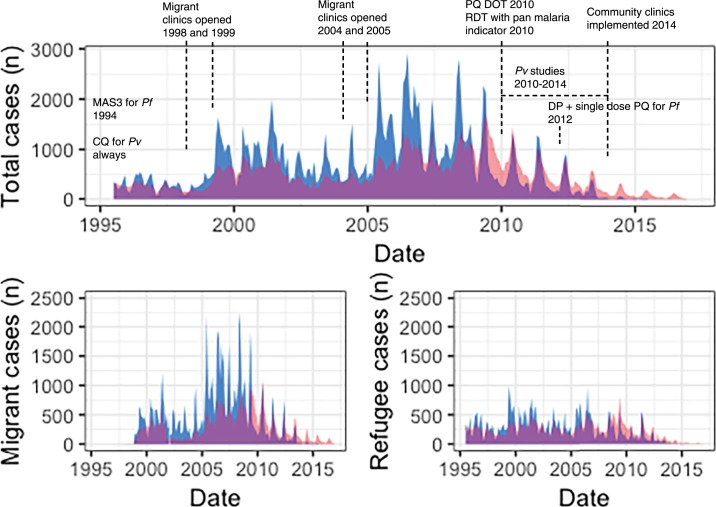
Number of cases of *Plasmodium falciparum* and *Plasmodium vivax* from 1995 to 2016 in the refugee and migrant clinics. *Plasmodium falciparum* is indicated in blue and *Plasmodium vivax* in red. CQ = chloroquine; DP = dihydroartemisinin piperaquine; MAS3 = mefloquine 25 mg/kg total dose plus artesunate 4 mg/kg/day for 3 days; PQ DOT = primaquine directly observed treatment (in patients who agreed to follow up); RDT = rapid diagnostic test.

**Figure 2.**
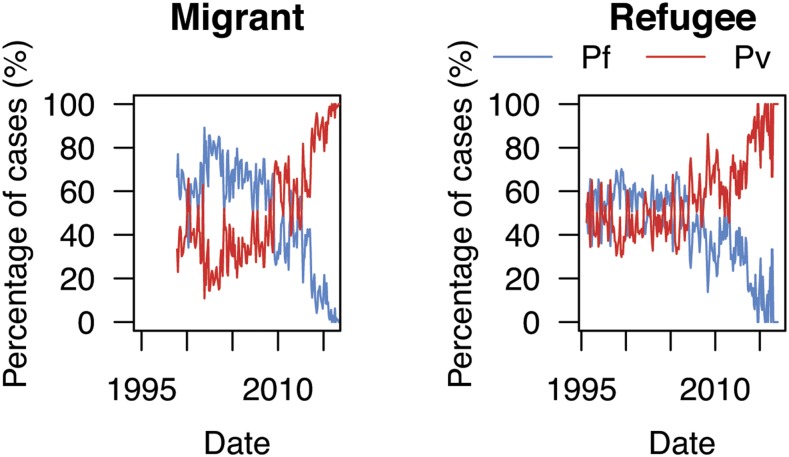
Percentage of *Plasmodium falciparum* and *Plasmodium vivax* for each year from 1998 to 2016 in migrant clinics and 1995 to 2016 in refugee clinics.

**Figure 3.**
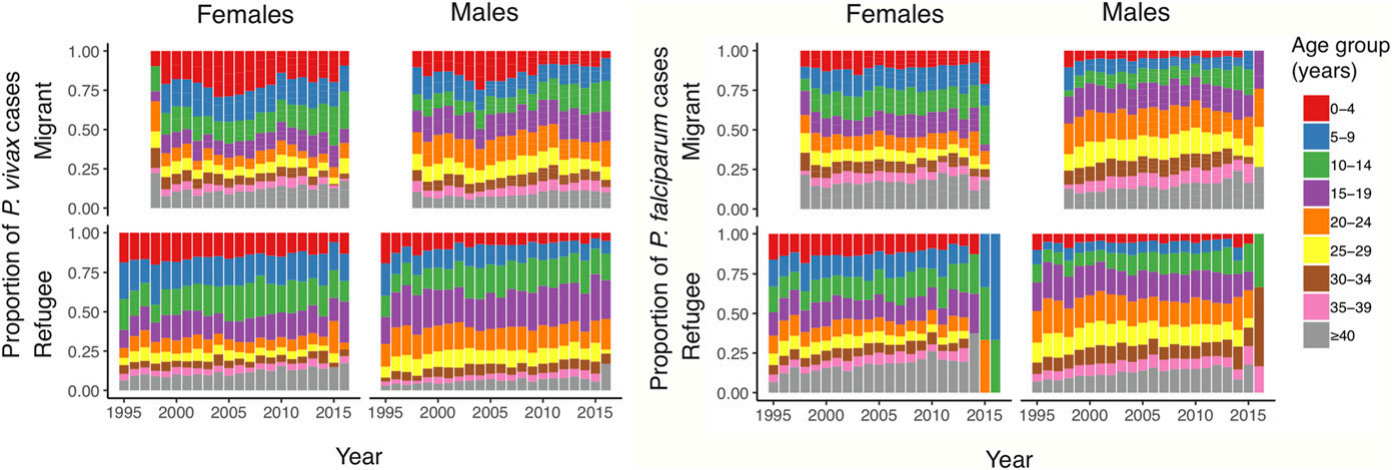
Annual proportions of *Plasmodium falciparum* and *Plasmodium vivax* by age and year, stratified by gender from 1998 to 2016 in migrant clinics and 1995 to 2016 in refugee clinics. In 2016, there were three refugee and no migrant female cases of *Plasmodium falciparum*.

Overall, the number of malaria cases decreased (Figure 1). Increases in 1999 and 2005 coincided with opening new migrant clinics and peaked in 2006. By the end of 2010, there was a 3-fold decrease in *P. falciparum* cases (from 2006 to 2010, 21,008 to 6,196 cases, respectively). *Plasmodium vivax* cases decreased nearly 2-fold, but the decline was delayed (from 2006 to 2011, 11,914 to 6,642 cases, respectively) in comparison with *P. falciparum* (Figure 1). During this time, *P. vivax* transitioned to become the dominant species^6^ (Figure 2). After 2010, malaria positivity continued to decrease for both species. This occurred alongside a ∼50% decline in consultations for non-malarial fever from 2011 to 2014 and which plateaued after 2014. In 2014, 2015, and 2016, approximately 3,400, 6,600, and 6,200 cases, respectively, of *P. vivax* were treated by the community-based malaria clinics; many located a few days travel inside Myanmar. Migrants had a 50% higher risk for malaria (incidence risk ratio (IRR) 1.5, 95% CI: 1.40–1.67; *P* < 0.001) than refugees. In both migrant and refugee clinics, the greatest declines in malaria incidence risk occurred in the < 10 year age-groups (Figure 3) and nearly an 80% reduction for *P. falciparum* (2006–2010) and 70% reduction for *P. vivax* (2006–2011) in the 0–4 years group. Females had a 56% lower incidence risk of malaria per year (IRR 0.44, 95% CI: 0.40–0.49; *P* < 0.001) (Supplemental Table 1). When assessing IRR differences between gender by age-group, males aged ≥ 10 years had a higher incidence risk of *P. falciparum* and *P. vivax* malaria each year. This risk difference ended with the ≥ 40 year age-group for *P. vivax* but persisted for *P. falciparum* (Supplemental Tables 2 and 3). The overall trends and risks were similar when the analysis was performed with and without correcting for clinical *P. vivax* diagnosis.

## DISCUSSION

As *P. falciparum* cases have decreased, a coincidental and delayed decrease in *P. vivax* has followed.^9,10^ Similar to other parts of Southeast Asia where *P. falciparum* is being eliminated, *P. vivax* becomes dominant, although not all countries report consistent declines in *P. vivax* caseload.^11^ Early diagnosis, efficacious ACTs, and single-dose primaquine contribute to lower *P. falciparum* transmission^12,13^; however, in the absence of widespread radical cure, the explanation for declining *P. vivax* is less clear.

The observed trend was not affected by the introduction of pan-malaria RDTs in 2010. It is also unlikely that the provision of radical cure to < 10% of *P. vivax* patients would drastically reduce the incidence risk of *P. vivax* as demonstrated here. A more probable explanation to the decreasing *P. vivax* cases at SMRU medical clinics is the opening of more than 1,200 community-based malaria clinics along the border, where similar shifts have been reported^14^; however, radical cure for *P. vivax* is not given there. It is also possible that reducing *P. falciparum* transmission reduces *P. vivax* infections mainly by decreasing relapse activation.^15,16^

Other factors potentially contribute to the decline of *P. vivax*. From 2009 to 2016, more than 280,000 insecticide-treated bed nets were distributed in the area, but their effectiveness is decreased by early evening mosquito biting behavior.^17^ Broad changes in the landscape along the international border, including deforestation, industrial agriculture, and urbanization,^18,19^ may contribute to an overall decrease in malaria, although a direct association cannot be made with these data. Gametocytocidal treatment with ACTs may contribute to decreased *P. vivax* transmission^20^; however, mixed infections are uncommon. A series of clinical studies (2010–2014) using radical curative primaquine may have contributed to decreased *P. vivax* transmission. This does not explain the longitudinal trends reported here or from the community clinics where no *P. vivax* studies are being conducted.^8^ Still, migrants and adult males remain at highest risk for *P. vivax* infection because of environmental and occupational exposure. Refugees (unless migratory) are confined to semi-urban camps where there is no malaria transmission. More detailed analyses stratified by age and gender are not possible with the available data.

In combination, species-specific diagnostics, bed nets, efficacious schizonticidal treatment, and comprehensive radical cure would presumably reduce the incidence of *P. vivax* at the same rate as *P. falciparum*. Without widespread radical cure, we postulate that the coincidental decline in *P. vivax* and its delay relative to *P. falciparum* in this area are due to decreased activation of *P. vivax* hypnozoites from reduced *P. falciparum* transmission.^15,16^ As the region approaches *P. falciparum* elimination, it is clear that *P. vivax* also requires a program for elimination. The trends reported here are promising, although radical cure will likely be necessary to completely eliminate *P. vivax* from this region.

## Supplemental tables

Supplemental materials
