# Supplementary material for: Declining Burden of Plasmodium vivax in a Population in Northwestern Thailand from 1995 to 2016 before Comprehensive Primaquine Prescription for Radical Cure
Source: Am J Trop Med Hyg. 2019 Nov 18;102(1):147–50. doi: 10.4269/ajtmh.19-0496 (PMC6947798; doi:10.4269/ajtmh.19-0496)
Supplement: Supplementary file 1 [file tpmd190496.SD1.pdf]

Supplement Table 1. Incidence Risk Ratio (IRR) for malaria overall, *Plasmodium falciparum, and Plasmodium vivax* in females

	IRR in males	IRR in females	95% CI	p-value
Malaria overall	Reference	0.44	0.40 to 0.49	<0.001
Plasmodium falciparum		0.37	0.34 to 0.41	<0.001
Plasmodium vivax		0.54	0.50 to 0.59	<0.001

Poisson regression was used to test for differences in trends over time, by age group and gender. Data were stratified by gender with an interaction term between year and age group.

Supplement Table 2. Incidence Risk Ratio (IRR) for *Plasmodium vivax* infection by age and time stratified by gender from 1995 to 2016 in refugee clinics and 1998 to 2016 in migrant clinics

Age group	Year	IRR refugee male	95% confidence interval	p-value	IRR refugee female	95% confidence interval	p-value	IRR migrant male	95% confidence interval	p-value	IRR migrant female	95% confidence interval	p-value
0-4 years	1995-1997	Reference			Reference								
	1998-2000	1.19	0.83 to 1.70	0.35	1.08	0.78 to 1.49	0.64	Reference			Reference		
	2001-2003	1.14	0.68 to 1.92	0.62	1.04	0.73 to 1.49	0.82	1.02	0.39 to 2.67	0.97	0.99	0.38 to 2.61	0.99
	2004-2006	0.94	0.59 to 1.50	0.79	0.91	0.64 to 1.31	0.62	3.16	1.21 to 8.20	0.02	3.15	1.23 to 8.05	0.02
	2007-2009	1.08	0.78 to 1.49	0.66	0.96	0.65 to 1.41	0.82	4.16	1.87 to 9.24	<0.01	4.40	1.97 yo 9.86	<0.01
	2010-2012	0.57	0.37 to 0.88	0.01	0.57	0.39 to 0.82	<0.01	1.31	0.52 to 3.34	0.57	1.45	0.59 to 3.56	0.42
	2013-2016	0.08	0.04 to 0.19	<0.01	0.09	0.03 to 0.25	<0.01	0.37	0.15 to 0.91	0.03	0.47	0.17 to 1.28	0.14
5-9 years	1995-1997	1.11	0.74 to 1.67	0.61	1.12	0.77 to 1.64	0.55						
	1998-2000	1.24	0.81 to 1.88	0.33	1.13	0.87 to 1.45	0.36	1.00	0.32 to 3.11	1.00	0.93	0.29 to 2.94	0.90
	2001-2003	1.61	1.09 to 2.38	0.02	1.14	0.84 to 1.54	0.40	0.89	0.32 to 2.48	0.83	0.90	0.32 to 2.55	0.84
	2004-2006	1.70	1.12 to 2.58	0.01	1.08	0.76 to 1.54	0.66	1.58	0.60 to 4.13	0.36	1.69	0.66 to 4.34	0.28
	2007-2009	1.67	1.14 to 2.44	0.01	1.17	0.79 to 1.73	0.43	2.82	1.27 to 6.28	0.01	2.87	1.28 to 6.44	0.01
	2010-2012	0.93	0.61 to 1.40	0.71	0.78	0.58 to 1.06	0.11	1.65	0.67 to 4.11	0.28	1.54	0.61 to 3.93	0.36
	2013-2016	0.13	0.06 to 0.28	<0.01	0.11	0.05 to 0.27	<0.01	0.52	0.20 to 1.36	0.18	0.46	0.17 to 1.22	0.12
10-14 years	1995-1997	1.05	0.66 to 1.67	0.83	0.96	0.70 to 1.32	0.81						
	1998-2000	1.40	0.89 to 2.22	0.15	0.89	0.56 to 1.42	0.64	0.70	0.23 to 2.15	0.53	0.80	0.25 to 2.59	0.71
	2001-2003	2.13	1.53 to 2.96	<0.01	1.15	0.87 to 1.51	0.34	0.54	0.17 to 1.73	0.30	0.66	0.22 to 1.99	0.46
	2004-2006	2.48	1.54 to 4.01	<0.01	1.27	0.82 to 1.98	0.28	1.48	0.59 to 3.70	0.40	1.31	0.52 to 3.31	0.57
	2007-2009	3.13	2.01 to 4.86	<0.01	1.58	1.10 to 2.26	0.01	2.32	1.03 to 5.18	0.04	2.01	0.89 to 4.52	0.09
	2010-2012	1.59	1.07 to 2.38	0.02	0.83	0.56 to 1.22	0.34	1.92	0.83 to 4.43	0.13	1.30	0.53 to 3.18	0.56
	2013-2016	0.29	0.13 to 0.67	<0.01	0.12	0.05 to 0.31	<0.01	0.69	0.27 to 1.73	0.43	0.52	0.21 to 1.31	0.17
15-19 years	1995-1997	1.85	1.06 to 3.25	0.03	0.77	0.53 to 1.12	0.17						
	1998-2000	2.53	1.66 to 3.86	<0.01	0.86	0.56 to 1.33	0.50	1.16	0.37 to 3.69	0.80	0.56	0.18 to 1.74	0.32
	2001-2003	2.76	1.85 to 4.12	<0.01	1.06	0.73 to 1.53	0.77	0.95	0.26 to 3.42	0.93	0.45	0.16 to 1.26	0.13
	2004-2006	2.62	1.74 to 3.97	<0.01	0.75	0.51 to 1.10	0.15	2.07	0.77 to 5.54	0.15	0.91	0.36 to 2.31	0.85
	2007-2009	3.77	2.59 to 5.49	<0.01	1.06	0.71 to 1.59	0.76	3.35	1.50 to 7.51	<0.01	1.69	0.75 to 3.79	0.21
	2010-2012	2.30	1.57 to 3.36	<0.01	0.70	0.49 to 1.00	0.05	2.36	0.98 to 5.70	0.06	1.04	0.41 to 2.62	0.93
	2013-2016	0.39	0.18 to 0.86	0.02	0.11	0.04 to 0.32	<0.01	0.77	0.29 to 2.08	0.61	0.35	0.13 to 0.98	0.05
20-24 years	1995-1997	1.46	0.84 to 2.54	0.18	0.53	0.33 to 0.84	0.01						
	1998-2000	1.72	1.09 to 2.71	0.02	0.48	0.32 to 0.73	<0.01	1.21	0.37 to 3.90	0.75	0.46	0.15 to 1.41	0.17
	2001-2003	2.08	1.38 to 3.14	<0.01	0.61	0.43 to 0.87	0.01	0.86	0.24 to 3.05	0.81	0.39	0.11 to 1.31	0.13
	2004-2006	1.68	1.05 to 2.70	0.03	0.40	0.29 to 0.56	<0.01	2.08	0.78 to 5.58	0.14	0.69	0.27 to 1.76	0.44
	2007-2009	2.31	1.63 to 3.29	<0.01	0.47	0.33 to 0.67	<0.01	3.37	1.51 to 7.53	<0.01	1.19	0.51 to 2.74	0.69
	2010-2012	1.29	0.87 to 1.92	0.21	0.32	0.23 to 0.44	<0.01	2.19	0.87 to 5.52	0.10	0.72	0.26 to 2.02	0.53
	2013-2016	0.20	0.09 to 0.45	<0.01	0.05	0.02 to 0.12	<0.01	0.50	0.19 to 1.29	0.15	0.18	0.07 to 0.48	<0.01
25-29 years	1995-1997	0.77	0.44 to 1.35	0.36	0.40	0.25 to 0.63	<0.01						
	1998-2000	1.03	0.68 to 1.56	0.90	0.33	0.24 to 0.46	<0.01	0.71	0.22 to 2.27	0.57	0.40	0.13 to 1.27	0.12
	2001-2003	1.45	0.98 to 2.14	0.06	0.40	0.30 to 0.53	<0.01	0.53	0.14 to 2.03	0.35	0.35	0.11 to 1.08	0.07
	2004-2006	1.13	0.70 to 1.82	0.62	0.32	0.24 to 0.42	<0.01	1.31	0.47 to 3.65	0.61	0.53	0.21 to 1.32	0.17
	2007-2009	1.45	1.04 to 2.03	0.03	0.34	0.25 to 0.45	<0.01	2.29	1.02 to 5.16	0.05	0.89	0.39 to 2.03	0.78
	2010-2012	0.86	0.57 to 1.31	0.48	0.21	0.14 to 0.30	<0.01	1.51	0.60 to 3.79	0.38	0.63	0.23 to 1.68	0.35
	2013-2016	0.15	0.06 to 0.38	<0.01	0.04	0.01 to 0.09	<0.01	0.34	0.12 to 1.02	0.05	0.15	0.06 to 0.38	<0.01
30-34 years	1995-1997	0.41	0.21 to 0.79	0.01	0.28	0.20 to 0.38	<0.01						
	1998-2000	0.57	0.32 to 1.04	0.07	0.29	0.19 to 0.45	<0.01	0.44	0.14 to 1.42	0.17	0.38	0.12 to 1.14	0.09
	2001-2003	0.88	0.58 to 1.32	0.53	0.30	0.23 to 0.39	<0.01	0.41	0.12 to 1.42	0.16	0.33	0.11 to 1.01	0.05
	2004-2006	0.82	0.56 to 1.20	0.30	0.30	0.24 to 0.39	<0.01	0.82	0.31 to 2.19	0.70	0.58	0.24 to 1.40	0.23
	2007-2009	0.85	0.57 to 1.28	0.44	0.31	0.20 to 0.47	<0.01	1.55	0.69 to 3.52	0.29	0.77	0.33 to 1.76	0.53
	2010-2012	0.56	0.38 to 0.82	<0.01	0.17	0.12 to 0.22	<0.01	1.11	0.45 to 2.73	0.83	0.54	0.21 to 1.41	0.21
	2013-2016	0.10	0.05 to 0.23	<0.01	0.04	0.01 to 0.09	<0.01	0.25	0.09 to 0.67	0.01	0.12	0.04 to 0.35	<0.01
35-39 years	1995-1997	0.21	0.13 to 0.36	<0.01	0.25	0.17 to 0.35	<0.01						
	1998-2000	0.35	0.18 to 0.67	<0.01	0.24	0.15 to 0.37	<0.01	0.32	0.10 to 1.02	0.05	0.24	0.07 to 0.76	0.02
	2001-2003	0.42	0.30 to 0.60	<0.01	0.28	0.18 to 0.44	<0.01	0.33	0.09 to 1.25	0.10	0.23	0.07 to 0.71	0.01
	2004-2006	0.50	0.26 to 0.96	0.04	0.29	0.18 to 0.45	<0.01	0.61	0.21 to 1.80	0.38	0.41	0.16 to 1.06	0.07
	2007-2009	0.63	0.43 to 0.94	0.02	0.27	0.16 to 0.46	<0.01	1.19	0.53 to 2.66	0.67	0.62	0.27 to 1.42	0.26
	2010-2012	0.38	0.28 to 0.52	<0.01	0.15	0.10 to 0.23	<0.01	0.83	0.33 to 2.12	0.70	0.52	0.21 to 1.28	0.15
	2013-2016	0.07	0.03 to 0.18	<0.01	0.04	0.01 to 0.11	<0.01	0.23	0.09 to 0.61	<0.01	0.12	0.04 to 0.34	<0.01
≥ 40 years	1995-1997	0.31	0.17 to 0.55	<0.01	0.51	0.33 to 0.81	<0.01						
	1998-2000	0.49	0.30 to 0.78	<0.01	0.54	0.37 to 0.79	<0.01	0.46	0.15 to 1.41	0.17	0.50	0.15 to 1.61	0.25
	2001-2003	0.71	0.51 to 0.98	0.04	0.64	0.48 to 0.87	<0.01	0.43	0.12 to 1.55	0.20	0.49	0.14 to 1.66	0.25
	2004-2006	0.83	0.55 to 1.24	0.37	0.63	0.44 to 0.90	0.01	0.96	0.37 to 2.48	0.93	0.94	0.37 to 2.38	0.90
	2007-2009	1.04	0.70 to 1.54	0.86	0.89	0.60 to 1.31	0.54	1.74	0.76 to 3.95	0.19	1.77	0.76 to 4.13	0.19
	2010-2012	0.70	0.47 to 1.02	0.07	0.60	0.43 to 0.84	<0.01	1.51	0.62 to 3.68	0.36	1.25	0.51 to 3.07	0.63
	2013-2016	0.13	0.06 to 0.30	<0.01	0.10	0.04 to 0.27	<0.01	0.44	0.17 to 1.17	0.10	0.38	0.14 to 0.99	0.05

Poisson regression was used to test for differences in trends over time, by age group, gender, and migratory status. Data were stratified by gender with an interaction term between year and age group.

Supplement Table 3. Incidence Risk Ratio (IRR) for *Plasmodium falciparum* infection by age and time stratified by gender from 1995 to 2016 in refugee clinics and 1998 to 2016 in migrant clinics

Age group	Year	IRR refugee male	95% confidence interval	p-value	IRR refugee female	95% confidence interval	p-value	IRR migrant male	95% confidence interval	p-value	IRR migrant female	95% confidence interval	p-value
0-4 years	1995-1997	Reference			Reference								
	1998-2000	1.45	1.10 to 1.89	0.01	1.53	1.03 to 2.28	0.03	Reference			Reference		
	2001-2003	1.23	0.96 to 1.59	0.11	1.37	0.95 to 1.98	0.09	1.24	0.57 to 2.70	0.58	1.43	0.63 to 3.23	0.39
	2004-2006	1.17	0.91 to 1.51	0.23	1.19	0.83 to 1.72	0.35	2.47	1.06 to 5.75	0.04	2.28	0.95 to 5.48	0.07
	2007-2009	0.74	0.56 to 0.98	0.03	0.65	0.43 to 0.97	0.04	1.96	0.92 to 4.17	0.08	2.05	0.93 to 4.53	0.08
	2010-2012	0.42	0.27 to 0.64	<0.01	0.30	0.16 to 0.56	<0.01	0.69	0.33 to 1.43	0.32	0.64	0.29 to 1.40	0.27
	2013-2016	0.03	0.01 to 0.07	<0.01	0.02	0.00 to 0.06	<0.01	0.07	0.01 to 0.34	<0.01	0.10	0.02 to 0.41	<0.01
5-9 years	1995-1997	0.97	0.77 to 1.21	0.78	1.02	0.71 to 1.46	0.91						
	1998-2000	1.63	1.14 to 2.33	0.01	1.66	1.05 to 2.63	0.03	1.35	0.48 to 3.80	0.57	1.15	0.39 to 3.43	0.80
	2001-2003	1.50	1.12 to 2.02	0.01	1.34	0.91 to 1.97	0.14	2.06	0.93 to 4.60	0.08	1.65	0.73 to 3.72	0.23
	2004-2006	1.73	1.31 to 2.28	<0.01	1.43	1.00 to 2.05	0.05	3.39	1.41 to 8.14	0.01	3.15	1.32 to 7.55	0.01
	2007-2009	1.08	0.76 to 1.53	0.68	0.76	0.52 to 1.10	0.14	3.62	1.70 to 7.70	<0.01	3.01	1.36 to 6.65	0.01
	2010-2012	0.66	0.42 to 1.02	0.06	0.52	0.28 to 0.95	0.04	1.16	0.55 to 2.47	0.69	1.02	0.45 to 2.31	0.97
	2013-2016	0.04	0.01 to 0.13	<0.01	0.03	0.01 to 0.09	<0.01	0.15	0.03 to 0.73	0.02	0.15	0.03 to 0.70	0.02
10-14 years	1995-1997	1.40	1.00 to 1.96	0.05	1.00	0.67 to 1.49	1.00						
	1998-2000	2.18	1.54 to 3.08	<0.01	1.49	0.89 to 2.49	0.13	1.27	0.43 to 3.77	0.66	1.24	0.42 to 3.69	0.70
	2001-2003	2.26	1.78 to 2.87	<0.01	1.37	0.95 to 1.97	0.10	1.86	0.81 to 4.28	0.14	1.65	0.70 to 3.89	0.26
	2004-2006	3.00	2.15 to 4.19	<0.01	1.43	0.96 to 2.15	0.08	4.54	1.90 to 10.8	<0.01	3.73	1.54 to 9.05	<0.01
	2007-2009	2.01	1.51 to 2.68	<0.01	1.01	0.71 to 1.44	0.95	4.78	2.24 to 10.2	<0.01	3.62	1.58 to 8.27	<0.01
	2010-2012	1.07	0.74 to 1.57	0.71	0.58	0.30 to 1.09	0.09	1.73	0.82 to 3.65	0.15	0.97	0.43 to 2.22	0.95
	2013-2016	0.11	0.03 to 0.43	<0.01	0.04	0.01 to 0.14	<0.01	0.21	0.05 to 0.92	0.04	0.13	0.03 to 0.59	0.01
15-19 years	1995-1997	3.62	2.14 to 6.13	<0.01	1.10	0.75 to 1.61	0.62						
	1998-2000	4.89	3.22 to 7.43	<0.01	1.66	0.98 to 2.81	0.06	2.90	1.01 to 8.38	0.05	1.06	0.37 to 3.06	0.91
	2001-2003	4.25	2.73 to 6.61	<0.01	1.42	0.89 to 2.27	0.15	3.86	1.70 to 8.79	<0.01	1.22	0.51 to 2.93	0.66
	2004-2006	4.14	3.00 to 5.70	<0.01	1.30	0.92 to 1.84	0.14	8.11	3.33 to 19.8	<0.01	3.01	1.20 to 7.53	0.02
	2007-2009	2.70	1.92 to 3.78	<0.01	0.83	0.54 to 1.27	0.39	8.24	3.63 to 18.7	<0.01	3.21	1.40 to 7.32	0.01
	2010-2012	1.73	1.12 to 2.67	0.01	0.45	0.33 to 0.62	<0.01	2.87	1.31 to 6.26	0.01	1.00	0.45 to 2.21	0.99
	2013-2016	0.12	0.03 to 0.47	<0.01	0.03	0.01 to 0.14	<0.01	0.28	0.06 to 1.24	0.09	0.11	0.02 to 0.49	<0.01
20-24 years	1995-1997	3.50	2.11 to 5.80	<0.01	0.93	0.60 to 1.42	0.72						
	1998-2000	4.55	2.93 to 7.06	<0.01	0.97	0.61 to 1.54	0.88	3.22	1.10 to 9.41	0.03	0.90	0.31 to 2.60	0.84
	2001-2003	4.49	3.01 to 6.69	<0.01	1.06	0.70 to 1.61	0.78	4.24	1.88 to 9.59	<0.01	0.98	0.40 to 2.41	0.97
	2004-2006	3.78	2.76 to 5.18	<0.01	0.86	0.60 to 1.24	0.42	9.01	3.53 to 23.0	<0.01	2.26	0.94 to 5.39	0.07
	2007-2009	2.32	1.77 to 3.05	<0.01	0.43	0.31 to 0.61	<0.01	9.28	4.12 to 20.9	<0.01	1.95	0.82 to 4.63	0.13
	2010-2012	1.44	0.94 to 2.20	0.10	0.31	0.20 to 0.45	<0.01	2.94	1.30 to 6.67	0.01	0.63	0.27 to 1.46	0.28
	2013-2016	0.09	0.03 to 0.26	<0.01	0.02	0.00 to 0.07	<0.01	0.24	0.05 to 1.04	0.06	0.06	0.02 to 0.23	<0.01
25-29 years	1995-1997	2.42	1.47 to 3.97	<0.01	0.63	0.39 to 1.03	0.07						
	1998-2000	3.46	2.13 to 5.61	<0.01	0.77	0.45 to 1.33	0.35	2.42	0.81 to 7.25	0.11	0.75	0.26 to 2.17	0.60
	2001-2003	3.64	2.42 to 5.47	<0.01	0.79	0.57 to 1.10	0.17	2.93	1.29 to 6.66	0.01	0.80	0.34 to 1.90	0.62
	2004-2006	3.09	2.25 to 4.24	<0.01	0.67	0.48 to 0.92	0.01	6.84	2.77 to 16.9	<0.01	1.83	0.75 to 4.48	0.19
	2007-2009	1.88	1.37 to 2.58	<0.01	0.31	0.19 to 0.51	<0.01	7.64	3.44 to 17.0	<0.01	1.67	0.74 to 3.76	0.22
	2010-2012	1.14	0.75 to 1.72	0.55	0.18	0.13 to 0.26	<0.01	2.51	1.10 to 5.71	0.03	0.43	0.17 to 1.06	0.07
	2013-2016	0.07	0.02 to 0.29	<0.01	0.01	0.00 to 0.06	<0.01	0.19	0.04 to 0.89	0.04	0.06	0.01 to 0.24	<0.01
30-34 years	1995-1997	1.38	0.83 to 2.29	0.22	0.46	0.31 to 0.67	<0.01						
	1998-2000	2.50	1.60 to 3.90	<0.01	0.68	0.40 to 1.15	0.15	1.60	0.55 to 4.63	0.38	0.65	0.23 to 1.85	0.42
	2001-2003	2.83	1.91 to 4.20	<0.01	0.66	0.46 to 0.94	0.02	2.47	1.06 to 5.74	0.04	0.78	0.33 to 1.85	0.57
	2004-2006	2.44	1.75 to 3.38	<0.01	0.66	0.48 to 0.89	0.01	4.96	2.03 to 12.1	<0.01	1.73	0.73 to 4.13	0.22
	2007-2009	1.32	0.91 to 1.91	0.14	0.39	0.21 to 0.73	<0.01	5.63	2.57 to 12.3	<0.01	1.42	0.63 to 3.21	0.40
	2010-2012	0.81	0.52 to 1.24	0.33	0.19	0.13 to 0.29	<0.01	1.85	0.85 to 4.04	0.12	0.46	0.20 to 1.05	0.07
	2013-2016	0.08	0.03 to 0.26	<0.01	0.01	0.00 to 0.06	<0.01	0.16	0.03 to 0.94	0.04	0.05	0.01 to 0.24	<0.01
35-39 years	1995-1997	0.92	0.55 to 1.53	0.75	0.36	0.23 to 0.55	<0.01						
	1998-2000	1.65	1.05 to 2.61	0.03	0.43	0.28 to 0.66	<0.01	1.03	0.34 to 3.14	0.95	0.46	0.15 to 1.37	0.16
	2001-2003	1.81	1.28 to 2.56	<0.01	0.60	0.42 to 0.84	<0.01	1.61	0.72 to 3.59	0.25	0.74	0.32 to 1.70	0.48
	2004-2006	1.85	1.23 to 2.77	<0.01	0.62	0.42 to 0.90	0.01	4.28	1.71 to 10.7	<0.01	1.45	0.59 to 3.57	0.42
	2007-2009	1.03	0.76 to 1.40	0.84	0.35	0.25 to 0.48	<0.01	4.17	1.92 to 9.05	<0.01	1.52	0.67 to 3.46	0.31
	2010-2012	0.63	0.37 yo 1.06	0.08	0.18	0.11 to 0.29	<0.01	1.73	0.79 to 3.75	0.17	0.44	0.19 to 0.98	0.05
	2013-2016	0.04	0.01 to 0.14	<0.01	0.02	0.00 to 0.10	<0.01	0.16	0.03 to 0.80	0.03	0.06	0.01 to 0.25	<0.01
≥ 40 years	1995-1997	1.30	0.78 to 2.18	0.31	0.89	0.51 to 1.52	0.66						
	1998-2000	2.58	1.63 to 4.08	<0.01	1.50	0.91 to 2.46	0.11	1.73	0.59 to 5.01	0.32	1.21	0.42 to 3.43	0.73
	2001-2003	2.94	2.09 to 4.12	<0.01	1.62	1.04 to 2.53	0.03	2.60	1.18 to 5.73	0.02	1.68	0.71 to 3.98	0.24
	2004-2006	3.59	2.40 to 5.38	<0.01	1.81	1.28 to 2.58	<0.01	7.00	2.82 to 17.4	<0.01	4.09	1.68 to 9.92	<0.01
	2007-2009	2.23	1.59 to 3.13	<0.01	1.15	0.79 to 1.68	0.46	7.22	3.32 to 15.7	<0.01	3.85	1.73 to 8.55	<0.01
	2010-2012	1.48	1.00 to 2.18	0.05	0.78	0.50 to 1.22	0.28	2.99	1.38 to 6.46	0.01	1.41	0.64 to 3.13	0.40
	2013-2016	0.10	0.02 to 0.41	<0.01	0.04	0.01 to 0.17	<0.01	0.40	0.09 to 1.85	0.24	0.17	0.03 to 0.86	0.03

Poisson regression was used to test for differences in trends over time, by age group, gender, and migratory status. Data were stratified by gender with an interaction term between year and age group.
